# Sleep disorders among healthcare staff at a university hospital in Tunisia

**DOI:** 10.1192/j.eurpsy.2026.12101

**Published:** 2026-07-31

**Authors:** A. Chouchane, Y. Tebourbi, W. Ben Abdejlil, I. Kacem, A. Aloui, A. Gaddour, S. Ben Fredj, M. Makhloufi, M. Bouhoula, A. Brahem, H. Kalboussi, O. ElMaalel, S. Chatti

**Affiliations:** 1 Occupational Medicine and Professional Pathologies Department, Farhat Hached Teaching Hospital; 2 Faculty of Medicine of Sousse, Research Laboratory LR19SP03, University of Sousse, Sousse; 3 Occupational Medicine and Professional Pathologies Department, Ibn El Jazzar teaching Hospital, Kairouan; 4 Department of Epidemilogy, Farhat Hached Teaching Hospital, Sousse, Tunisia

## Abstract

**Introduction:**

Healthcare professionals work in a highly demanding environment characterized by long working hours, significant psychological stress, and frequent shift work. These occupational factors place them at a high risk for developing sleep disorders.

**Objectives:**

The aim of this study was to assess the prevalence of excessive daytime sleepiness, a key indicator of underlying sleep disorders, among healthcare staff at a university hospital in Tunisia.

**Methods:**

A cross-sectional study was conducted from May to August 2023 among 300 permanent healthcare staff at the Farhat Hached University Hospital in Sousse. Data on socio-demographic, professional, and lifestyle characteristics were collected via face-to-face interviews using a pre-established questionnaire. Daytime sleepiness was assessed using the Epworth Sleepiness Scale.

**Results:**

The study population was predominantly female (80%) with a mean age of 43.6±10.2 years. Nurses constituted the largest professional group (40.3%) and most participants (70.3%) worked fixed hours. The average length of professional experience was 15.8±10.9 years. Sleep disorders were reported by 39.7% (n=119) of the participants. The mean sleep duration was 6.6 ± 1.3 hours (range: 3-12 hours). Excessive daytime sleepiness, defined as an Epworth Scale score >10, was present in 22.3% (n=67) of healthcare staff, with a mean Epworth score of 7.5 ± 3.8.

**Conclusions:**

This study reveals a significant prevalence of sleep disorders and excessive daytime sleepiness among healthcare staff. With nearly one in four staff members reporting levels of sleepiness that could impair cognitive function and performance, these findings highlight a critical issue for both staff well-being and patient safety and underscore the need for targeted occupational health interventions such as sleep hygiene education.

**Disclosure of Interest:**

None Declared

